# Comparative analysis of protein interactome networks prioritizes candidate genes with cancer signatures

**DOI:** 10.18632/oncotarget.12879

**Published:** 2016-10-25

**Authors:** Yongsheng Li, Nidhi Sahni, Song Yi

**Affiliations:** ^1^ Department of Systems Biology, The University of Texas MD Anderson Cancer Center, Houston, TX 77030, USA; ^2^ Graduate Program in Structural and Computational Biology and Molecular Biophysics, Baylor College of Medicine, Houston, TX 77030, USA

**Keywords:** comparative network analysis, protein interaction networks, prioritization of cancer genes, network centrality, systems biology

## Abstract

Comprehensive understanding of human cancer mechanisms requires the identification of a thorough list of cancer-associated genes, which could serve as biomarkers for diagnoses and therapies in various types of cancer. Although substantial progress has been made in functional studies to uncover genes involved in cancer, these efforts are often time-consuming and costly. Therefore, it remains challenging to comprehensively identify cancer candidate genes. Network-based methods have accelerated this process through the analysis of complex molecular interactions in the cell. However, the extent to which various interactome networks can contribute to prediction of candidate genes responsible for cancer is still enigmatic. In this study, we evaluated different human protein-protein interactome networks and compared their application to cancer gene prioritization. Our results indicate that network analyses can increase the power to identify novel cancer genes. In particular, such predictive power can be enhanced with the use of unbiased systematic protein interaction maps for cancer gene prioritization. Functional analysis reveals that the top ranked genes from network predictions co-occur often with cancer-related terms in literature, and further, these candidate genes are indeed frequently mutated across cancers. Finally, our study suggests that integrating interactome networks with other omics datasets could provide novel insights into cancer-associated genes and underlying molecular mechanisms.

## INTRODUCTION

Over the past few decades, cancer related genes have generally been identified using genome wide association studies [1–3]. Although large numbers of cancer related gene candidates have been discovered [4, 5], it is still difficult to understand the mechanisms underlying cancer. Given the functional interdependencies among genes in a human cell, it is well known that cancer is rarely a consequence of an abnormality in a single gene but the perturbation of complex molecular networks [6].

With the release of large-scale protein-protein interaction (PPI) networks in human, the emerging of network medicine offers a platform to explore the molecular mechanisms in cancer [7–10]. Network analyses have indicated that highly connected nodes in protein interaction networks, called hubs, have a special biological role. It has been demonstrated that genes encoding hubs are often conserved and essential [11]. These results have led to the hypothesis that, in humans, hub genes could be associated with cancer [12]. Based on this hypothesis, an increasing number of studies have begun to use interactome networks to prioritize cancer related genes [13–16]. The topological features of degree and other centrality are demonstrated to be valuable measures for predicting disease genes [17]. Using a phenomic ranking of protein complexes linked to human disease, Lage et al. (2007) have developed a Bayesian predictor to identify disease related genes by pooling human interaction data from several large databases [18]. Xu and Li (2006) have used a classifier based on several topological features, including degree, to identify cancer genes. In their study, Online Predicted Human Interaction Database (OPID) was used [14]. Although these studies have already provided network-based methods to identify cancer genes, the networks used in these studies remain incomplete and insufficient quality to derive accurate global prioritization.

With the development of high-throughput experimental and large-scale computational methods, systematic interactome datasets in human were generated. Zhang et al. (2013) generated proteome-scale interactions among genes based on computational predictions (PrePPI) [19]. However, this interactome dataset might be inherently limited by biases of current knowledge used in prediction models. In addition, by applying an integrative global proteomic profiling approach, Havugimana et al. identified a network (Co-Frac) of 13,993 high-confidence physical interactions among 3,006 human proteins [20]. Recently, Huttlin et al. generated a proteome-scale interactome dataset in human using high-throughput affinity-purification mass spectrometry (AP-MS), resulting in 23,744 interactions among 7,688 proteins [21]. In addition, Rolland et al. reported a systematic unbiased map of about 14,000 high-quality human binary PPIs (HI-II-14) as well as an interactome dataset composed of curated PPIs with at least two pieces of supporting evidence in literature (Lit-BM) [22]. These proteome-scale interactome datasets will enable deeper insights into understanding genotype-phenotype relationships in human disease.

Given that the majority of existing cancer gene prioritization studies are based on literature knowledge, the comprehensive list of cancer genes remains to be discovered. With the emergence of systematic proteome-scale interactome mapping technologies, it provides a unique opportunity not only to identify novel cancer candidate genes, but also to comparatively evaluate the power of each interactome network map for prioritization of cancer genes. Here, we systematically analyzed multiple PPI network datasets and compared their predictive power for cancer candidate gene identification.

## RESULTS

### Comparative analysis of interactome network datasets for prioritization of cancer genes

**Figure 1:**
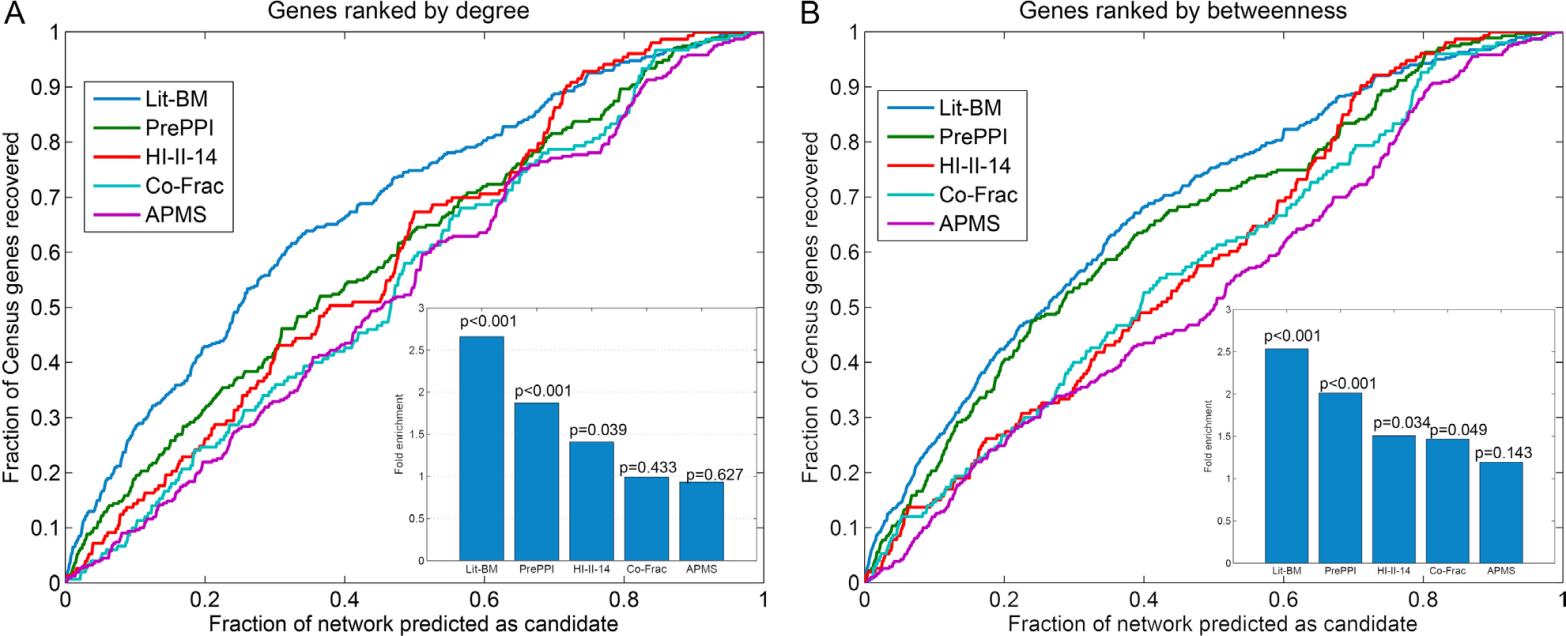
Comparative analysis of different interactome datasets for prioritizing cancer genes (**A**) The recall rate of different interactome datasets based on degree. (**B**) The recall rate of different interactome datasets based on betweenness. The figures on the bottom right are fold enrichment of each network at the cutoff of 10%. *P*-values are computed based on the frequencies of occurrence in randomized networks.

Human PPI networks have been widely used in prioritizing cancer related genes. To investigate the power of different interactome datasets for prioritizing cancer genes, we assembled five types of human PPI networks from previous studies (see methods). We then ranked each gene based on their topological features in each network. Using the genes obtained from Sanger Cancer Gene Census (CGC) as gold standard for known cancer genes, we found that the literature-based network (Lit-BM) exhibited the highest power to recover known cancer genes when genes were ranked by degree (Figure 1A and 1B). In addition, the PrePPI network also recovered known cancer genes effectively. It was expected that Lit-BM and PrePPI had higher recovery than the other three types of systematic experimental networks, since the former two were both based on the literature. Use of these two networks for prioritizing cancer genes might be restricted to genes with multiple pieces of evidence in the literature.

**Figure 2:**
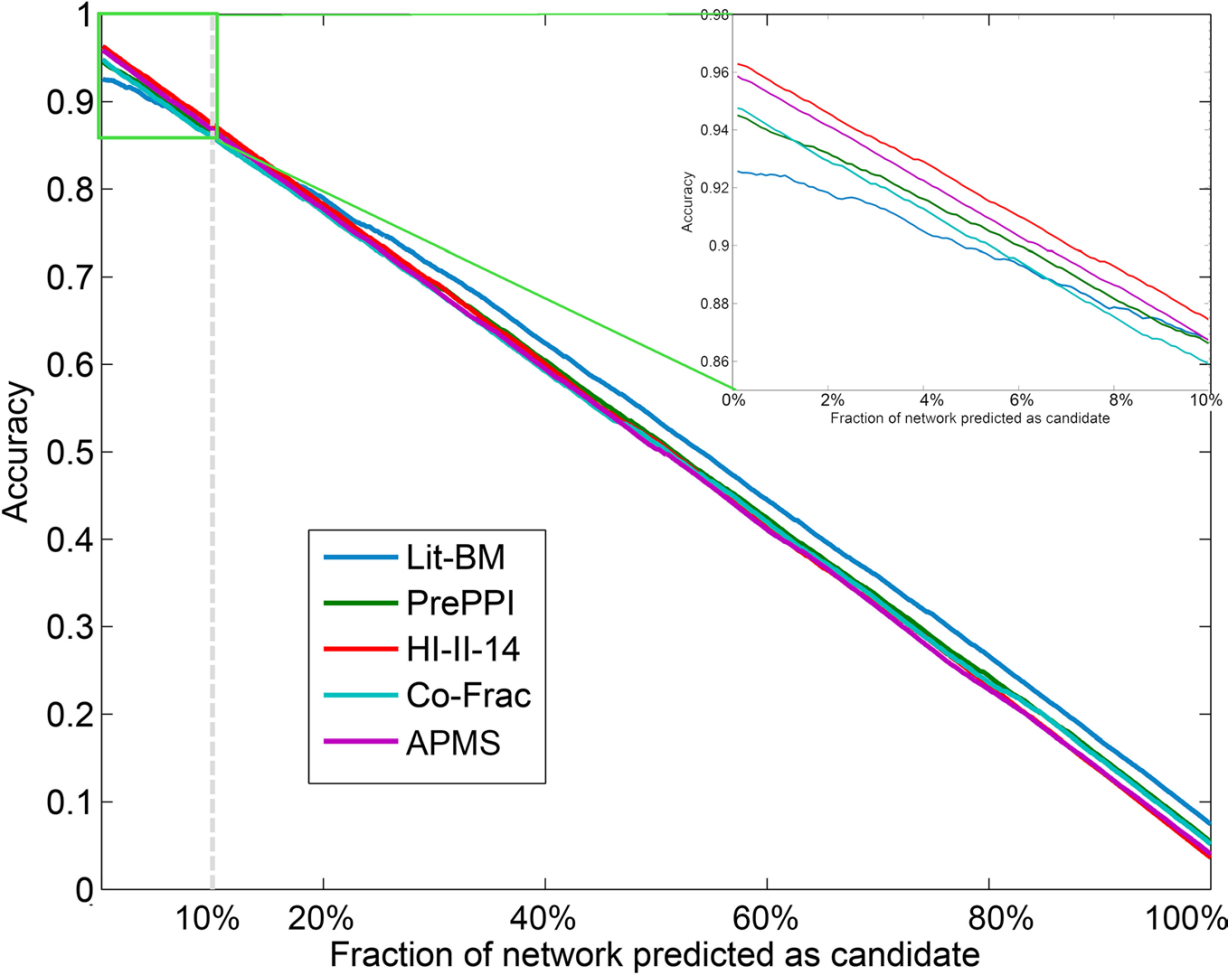
The accuracy of different interactome networks in prioritizing cancer genes

Recently, several lines of evidence have demonstrated that high-throughput systematic networks can generate novel interactions not found in literature. In terms of the systematic networks, HI-II-14, co-Frac and AP-MS all provide us a chance to identify cancer related genes in an unbiased manner. For these three types of systematic networks, it seemed that HI-II-14 exhibited a better performance than the other two (Figure 1A and 1B) in its predictive power. Next, we investigated whether “topological features” based method could effectively recover known cancer genes in each network context. Genes in each network were randomly ranked and then we computed the recovery for the top ranked 10% genes. This procedure was repeated 1,000 times. The fold enrichment was defined as the proportion of recovered genes based on network centrality ranking versus the mean proportion in random networks. As a result, we found that Lit-BM, PrePPI and HI-II-14 exhibited a strong tendency to recover known cancer genes (Figure 1A and 1B). However, the other two networks presented less power to recover these genes. Next, we also compared the accuracy of each network in predicting cancer genes, and found that HI-II-14 exhibited the highest accuracy compared to the other networks (Figure 2). Together, results from our comparative analyses suggest that HI-II-14 provides a high-quality and unique predictive power for prioritizing cancer genes from genome-wide studies.

### High-throughput systematic networks are robust for prioritization of cancer genes

**Figure 3:**
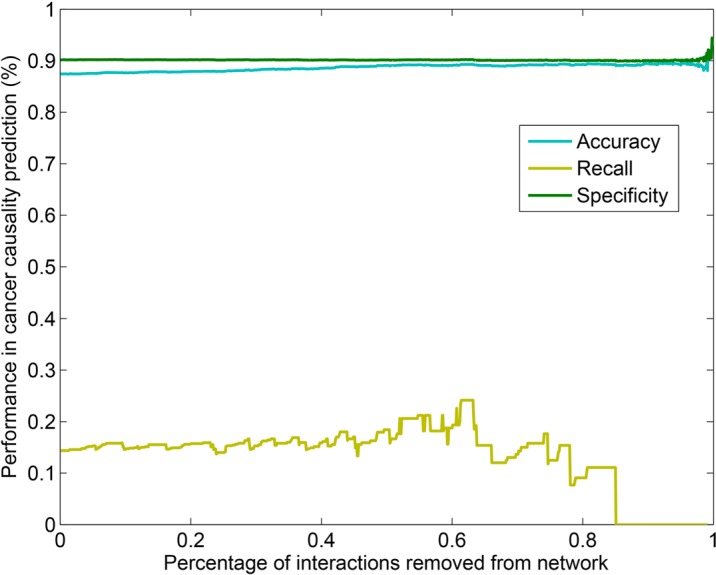
The accuracy, recall and specificity remain steady over a wide range of network sizes in the systematic unbiased HI-II-14 interactome

Despite advances in high-throughput systematic interactome mapping, the human interactome maps remain incomplete. Next, we investigated whether the incompleteness could affect the prediction of cancer genes. The results above indicate that HI-II-14 provided the best context for prioritizing cancer genes among systematic unbiased networks. Therefore, we focused on this network for further analysis. Increasing fractions of interactions were randomly removed from the original HI-II-14 network, and then we re-computed the degree of each gene. Genes were ranked by their newly computed degree and the recall, accuracy and specificity were obtained at different percentages of random interaction loss. By this down-sampling analysis, we found negligible effect on cancer gene prediction over a broad range of network sizes (Figure 3). These measurements, such as accuracy and specificity, remained steady over a wide range of HI-II-14 network sizes. These results suggest that despite the incompleteness of current high-throughput systematic networks, they have reached sufficient coverage to allow for accurate prioritization of cancer genes.

### Top ranked genes are involved in cancer related functions

**Figure 4:**
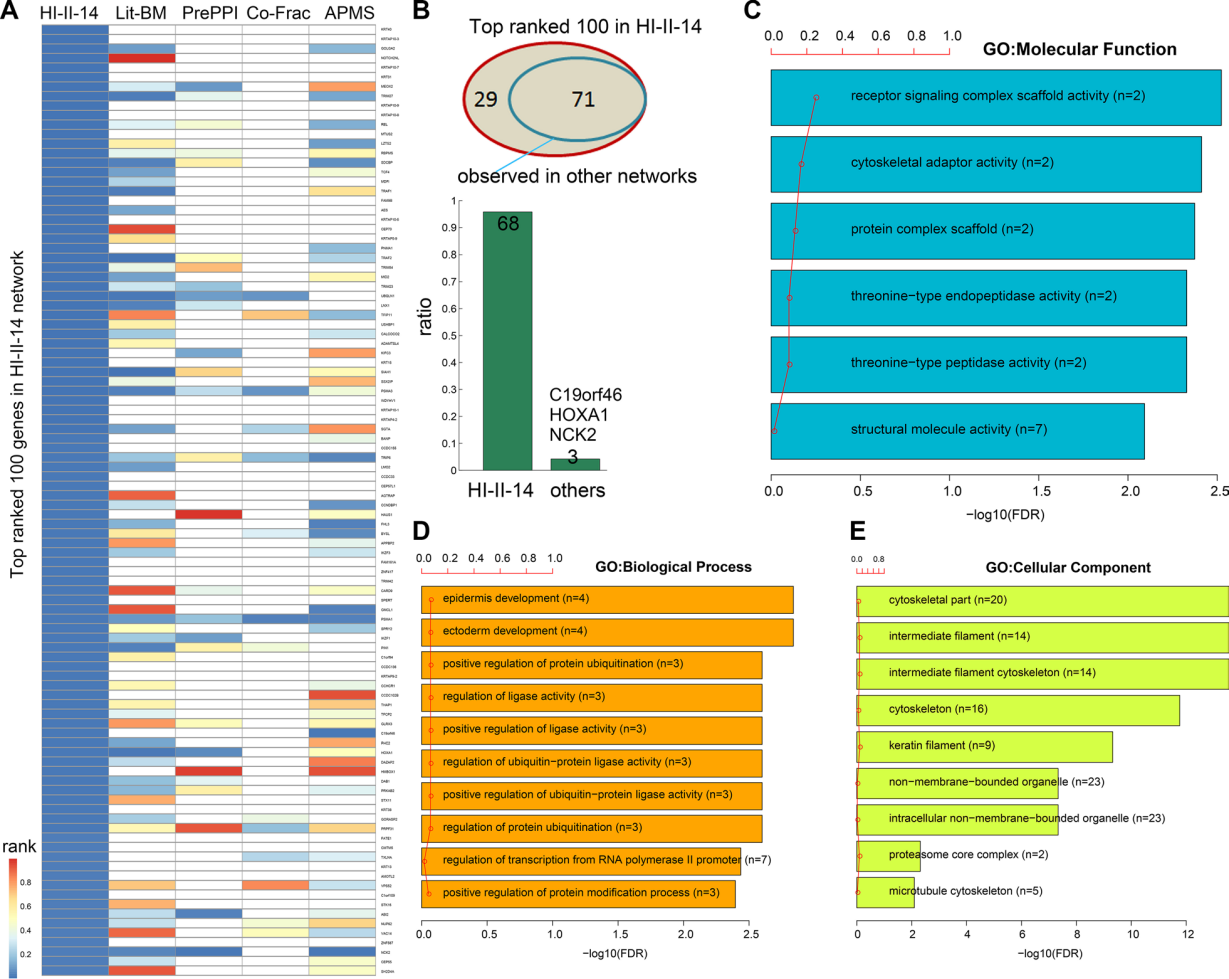
Functional enrichment of top ranked candidate genes in the systematic HI-II-14 network (**A**) Relative rank of top genes in each network. (**B**) Overlap of the top ranked genes between HI-II-14 network and other networks. (**C–E**) GO function enrichment for the top ranked 100 genes. Red points indicated the ratio of selected genes for each functional term.

Next, we focused on the top-100 ranked candidate cancer genes in HI-II-14 network. Of these genes, we first computed the relative rank in each network, which is defined as the degree rank divided by the total number of genes in the corresponding network. We observed that the majority of these 100 genes are with relatively lower rank in the HI-II-14 network (Figure 4A). There were 71 genes that were also observed in one of the other networks. However, only three genes (C19orf46, HOXA1 and NCK2) were with relatively lower rank in other networks (Figure 4B).

In addition, we investigated what functions these top ranked genes might play in cellular signaling. Functional enrichment analysis indicated that the majority of these genes were involved in regulation of enzyme activity functions (Figure 4C–4E), such as regulation of ligase activity and regulation of protein ubiquitination. Protein ubiquitination has been demonstrated in many cellular functions, such as cell proliferation, apoptosis, cell cycle regulation and DNA repair. It has become apparent that dys-regulation of ubiquitination pathways results in the development of many human diseases, including cancer [23].

### Top ranked genes are with higher mutation frequency across cancers

**Figure 5:**
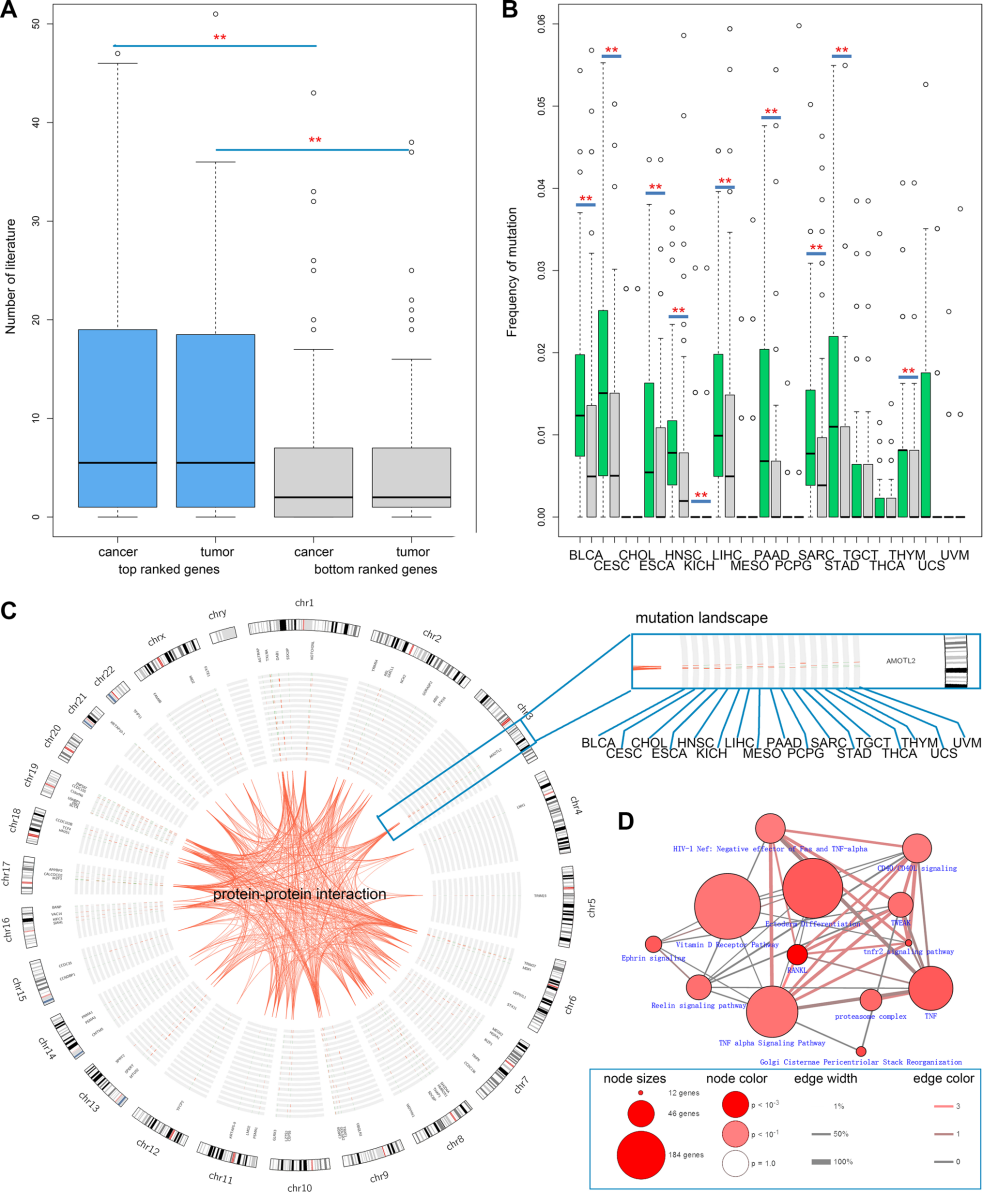
Top ranked predicted cancer candidate genes exhibit higher mutation frequency across cancers (**A**) Top ranked genes tend to co-occur with the key words “cancer” and “tumor” in literature. (**B**) Top ranked genes are with higher mutation frequency.(**C**) The circos plot shows the mutation landscape of the top ranked genes across 17 types of cancer. (**D**) The enriched pathways of top ranked genes.

In order to further explore whether the top ranked genes were related to cancer, we searched PubMed and counted the number of publications in which the gene and “cancer” or “tumor” co-occurred. We found that the top ranked genes more frequently co-occurred with cancer or tumor than the bottom ranked genes (Figure 5A, *p*-values < 0.01). In addition, we also computed the mutation frequency of the top ranked genes across 17 types of cancer in TCGA. We found that these genes were with higher mutation frequency in the majority of cancers (Figure 5B). All of the 100 top ranked genes were frequently mutated in at least one cancer sample (Figure 5C). These results suggested that the top ranked genes could be putative cancer drivers. Next, we performed pathway enrichment analysis for these genes using ConsensusPathDB [24, 25]. These genes were mainly involved in TNF signaling pathway (Figure 5D). TNF (Tumor Necrosis Factor) has been shown to be a multifunctional pro-inflammatory cytokine, with effects on lipid metabolism, coagulation, insulin resistance, as well as endothelial function [26–28]. Several lines of evidence have shown that TNF can be considered as an anti-cancer agent. Together, all these results strongly suggest that our prioritized candidates are likely to be cancer-associated genes.

## DISCUSSION

The identification of candidate driver cancer genes is important to researchers and clinicians for diagnosing and treating cancer. However, comprehensively identifying candidate cancer genes is time consuming and costly. Network based methods have accelerated the understanding of the mechanism underlying cancer. However, it is difficult to decide the optimal and appropriate network to use in this process. The purpose of this study was to evaluate different human interactome networks for their application to cancer gene prioritization. In order to evaluate the power of different interactome datasets, we used network centrality to rank genes in this study. Although other well-established disease gene prioritization methods like DAPPLE [29], Metaranker [30], PRINCE [31] and some random walk methods [32, 33] have been demonstrated to be valuable methods for predicting disease related genes, it is complex to compare these different methods in multiple network contexts.

A comparative analysis of five human interactome datasets, including Lit-BM, PrePPI, HI-II-14, co-Frac and AP-MS was performed. Our comparative analysis results indicate that these networks can be used to identify cancer related genes. In addition, Lit-BM, PrePPI and HI-II-14 may be good choices for this purpose. It is not surprising that Lit-BM and PrePPI network have higher power for the prioritization of cancer genes, because these two networks may have bias for the genes that are well studied. As shown in our previous study, interactions between highly studied genes formed a striking “dense zone” in contrast to a large sparse zone involving poorly studied genes [22]. These results emphasize a need for unbiased systematic PPI mapping for cancer gene prioritization. HI-II-14, co-Frac and AP-MS networks provide proteome-wide interactome datasets for cancer gene identification. Our comparative analysis indicates that HI-II-14 exhibits higher power for cancer gene prioritization than the other two systematic interactome networks.

Although most of these networks are currently incomplete, our network sampling analysis indicates that HI-II-14 network is robust to identify cancer related genes. In addition, we found that different networks only cover a subset of genes in the genome. However, for the top 100 ranked genes in HI-II-14, there are 71 genes also covered by other networks. Only three genes are with relatively lower rank in other networks, indicating the greater power of HI-II-14. Based on the HI-II-14 network, we ranked the genes by their degree. Functional analysis indicates that the top ranked genes co-occurred with cancer related terms frequently in literature. Furthermore, these genes were frequently mutated across cancers. Together, these results demonstrate the key roles of these top ranked genes in cancer.

Integration of multiple omics datasets, such as gene expression, mutation, functional annotation as well as molecular interaction datasets will lead to more accurate prediction of cancer genes. Our analysis indicates that high-throughput systematic networks such as HI-II-14 are valuable for cancer gene prioritization, and integrating such interaction networks with other omics datasets will undoubtedly provide novel insights into molecular mechanisms underlying cancer.

## MATERIALS AND METHODS

### Human interactome datasets

Here, five types of human protein-protein interaction (PPI) datasets were assembled from different databases and references. The following PPI networks were included for our analysis: the largest experimentally determined binary interaction map (HI-II-14) [22], a set of protein interactions with multiple pieces of evidence in the literature (Lit-BM) [22], a collection of predicted PPIs of high confidence (PrePPI) [3] and a systematic co-fractionation map (co-Frac) [24]. We also obtained the BioPlex (biophysical interactions of ORFeome-based complexes) network from the study of Huttlin et al., including 23,744 interactions among 7,668 genes [23]. All these interactions were mapped to NCBI Entrez gene ID and excluded self-loop and redundant interactions. The resulting statistics of interactions was summarized in Table 1.

**Table 1:** The statistics of different PPI resources

Network	#genes	#interactions	#cancer genes
Lit-BM	5,545	11,045	131
PrePPI	4,989	25,403	95
HI-II-14	4,303	13,944	45
Co-Frac	2,898	13,571	35
AP-MS	7,668	23,744	102

### Cancer genes

The known human cancer genes were obtained from the Sanger Cancer Gene Census (CGC) [34] which is a comprehensive catalogue of genes implicated in cancer. In total, we obtained 570 genes from CGC for analyses.

### Topological features of genes in protein-protein interaction networks

Here, we considered two types of topological features of genes in human protein-protein interaction networks. The first one is degree which is defined as the number of partners in the network. And the second one is betweenness centrality, which indicates how central a gene is in interaction networks. The betweenness is defined as

$${\text{BC}}_{\text{i}}=\sum_{\text{j}<k}\frac{{\text{g}}_{\text{jk}}(\text{i})}{{\text{g}}_{\text{jk}}}$$

where gjk(i) is the number of shortest paths between gene j and k across gene i, and is the total number of shortest paths that connect genes j and k. The higher of these two topological features, the more important the gene is in the network.

### Comparative analysis of different interactome networks

In order to compare the power of different interactome datasets in prioritizing cancer genes, we obtained known cancer genes from CGC. These genes were used as positive controls. The remaining genes in each network were considered as negative controls. Then the genes in each network were ranked based on the topological features (such as degree). For a selected threshold (for example top 10% genes ranked by topological features), we considered these top ranked genes as predicted cancer genes, and then the following measures were calculated:

$$\begin{array}{c} \text{recall}=\frac{\text{TP}}{\left(\text{TP}+\text{FN}\right)}\\ \text{accuracy}=\frac{\left(\text{TP}+\text{TN}\right)}{\left(\text{TP}+\text{FP}+\text{TN}+\text{FN}\right)}\\ \text{specificity}=\frac{\text{TN}}{\left(\text{TN}+\text{FP}\right)} \end{array}$$

Then, we computed these measures for all thresholds and compared the power of each network.

### Effect of network completeness

To evaluate the effect of network completeness on the prediction of cancer genes, we randomly removed increasing percentage of interactions from the PPI network, and re-predicted the cancer genes based on their topological features in the remaining network. We recalculated the recall, accuracy, and specificity for the performance as a function of fractions of random PPI loss.

### Genome-wide mutation datasets across cancers

The genome-wide mutation datasets across 17 types of cancer were downloaded from TCGA as maf files [35, 36]. Only the Hi-Seq platform datasets were considered in our analysis. For each gene, we obtained the mutation frequency which is defined as the number of mutated samples divided by the total number of samples in each cancer. The frequency of the top 100 ranked genes and the bottom 100 ranked genes were compared by Wilcox rank sum test.
